# Liraglutide Attenuates Hepatic Oxidative Stress, Inflammation, and Apoptosis in Streptozotocin-Induced Diabetic Mice by Modulating the Wnt/*β*-Catenin Signaling Pathway

**DOI:** 10.1155/2023/8974960

**Published:** 2023-01-29

**Authors:** Jie Yu, Yuan Zhao, Lingling Xu, Wei Li, Huabing Zhang, Fan Ping, Yuxiu Li

**Affiliations:** ^1^Key Laboratory of Endocrinology of National Health Commission, Department of Endocrinology, Peking Union Medical College Hospital, Chinese Academy of Medical Sciences & Peking Union Medical College, Beijing, China; ^2^Department of Endocrinology and Metabolism, Shanxi Medical University Second Hospital, Shanxi Medical University, China

## Abstract

Liraglutide has been extensively applied in the treatment of type 2 diabetes mellitus and also has hepatoprotective effects. However, the role of liraglutide treatment on liver injury in a mouse model of type 1 diabetes mellitus (T1DM) induced by streptozotocin (STZ) and its underlying mechanisms remain to be elucidated. In the present study, diabetes was initiated in experimental animals by single-dose intraperitoneal inoculation of STZ. Forty female C57BL/6J mice were equally assigned into five groups: diabetic group, insulin+diabetic group, liraglutide+diabetic group, insulin+liraglutide+diabetic group, and control group for eight weeks. Diabetic mice exhibited a significantly elevated blood glucose level and decreased body weight, and morphological changes of increased steatosis and apoptosis were observed in the liver compared with the control. Furthermore, a significant increase in the levels of malondialdehyde and inflammatory markers such as tumor necrosis factor-*α* (TNF-*α*), interleukin-6 (IL-6), and interleukin 1*β* (IL-1*β*) and the proapoptotic proteins caspase-3 and Bax were observed in the livers of diabetic mice, together with marked increases in antioxidants superoxide dismutase (SOD) and glutathione peroxidase (GPX) as well as antiapoptotic protein Bcl-2, all of which were significantly mitigated by treatment with liraglutide, insulin, and their combinations. Interestingly, liraglutide monotherapy showed better efficacy in ameliorating liver injury in T1DM mice than insulin monotherapy, similar to the combined drug therapy. Furthermore, the expression of Wnt/*β*-catenin signaling pathway-associated molecules was upregulated in the liver of mice treated with liraglutide or insulin. The results of the present study suggested that liraglutide improves T1DM-induced liver injury and may have important implications for the treatment of nonalcoholic fatty liver disease (NAFLD) in patients with T1DM.

## 1. Introduction

The relationship between diabetes and liver disease has been extensively studied and described, but mainly in type 2 diabetes mellitus (T2DM). Over the last few years, it has been reported that patients with type 1 diabetes mellitus (T1DM) may also be at increased risk of developing nonalcoholic fatty liver disease (NAFLD), possibly related to hyperglycemia-induced oxidative stress and apoptosis [1, 2], but also due to conditions specific to T1DM that may propel metabolic dysfunction [3, 4]. However, there is still a lack of effective treatments for NAFLD in patients with T1DM due to incomplete knowledge of the pathogenesis.

The development of NAFLD may involve multiple pathways, including metabolic disorders and functional abnormalities, leading to inflammation, apoptosis, and changes in antioxidant status. The glucagon-like peptide 1 (GLP-1) agonist liraglutide has been reported to improve hepatic metabolic dysfunction and inflammation and reduce hepatic lipid build-up in patients with NAFLD [5, 6]. Previous studies in animal models demonstrated that liraglutide improved liver inflammation in mice with high-fat diet-induced NAFLD [7], enhanced the antioxidant activity of liver cells, and decreased glucolipotoxicity-induced liver cell apoptosis in Zucker diabetic fatty rats [8]. However, it has not been evaluated whether liraglutide improves liver damage in individuals with T1DM.

The Wnt/*β*-catenin signaling pathway plays an important regulatory role in cell proliferation, differentiation, development, and metabolism [9, 10], and its activation was associated with the repair of fatty liver injury or promotion of liver regeneration in NAFLD [11]. Activation of Wnt/*β*-catenin signaling was also proved to improve hepatic steatosis, inflammatory response, and apoptosis in rats with alcoholic liver disease [12] and promoted hepatocyte proliferation and alleviated apoptosis in a model of liver ischemia-reperfusion injury [13]. Furthermore, liraglutide has been demonstrated to improve hepatic insulin resistance by directly activating the Wnt/*β*-catenin signaling pathway in mice with T2DM [14]. Therefore, this study is aimed at evaluating the effects of liraglutide, insulin, and the combined drugs on liver injury in STZ-induced T1DM mice and the underlying mechanisms.

## 2. Materials and Methods

### 2.1. Animals and Study Design

Forty female C57BL/6J mice of 10-11 weeks of age purchased from Beijing Huafukang Co., Ltd. (Beijing, China) were housed under standard laboratory conditions (air-conditioned atmosphere, 12 h light and dark cycle, humidity of 40-60%, and room temperature at 22°C) with free access to food and water. Animals were acclimated to laboratory conditions for one week before the start of the experiment. T1DM was induced via intraperitoneal injection of STZ (150 mg/kg body weight, once), and mice in the control group (NC) (*n* = 8) were injected with citrate buffer (100 mM citrate, pH 4.2-4.5).

Two weeks later, mice injected with STZ and with random blood glucose ≥ 16.7 mmol/l were randomly assigned to four treatment groups for 8 weeks (*n* = 8 in each group): (1) diabetic group (DM), subcutaneously injected with normal saline; (2) diabetic+insulin group (DMI), subcutaneously injected with insulin (10 units/kg body weight/day, as detemir insulin, Levemir®, Novo Nordisk, Denmark); (3) diabetic+liraglutide group (DML), subcutaneously injected with liraglutide (0.6 mg/kg/day, Novo Nordisk, Denmark), which referred to the insulin dose used in the previous study [15]; and (4) diabetic+insulin+liraglutide group (DMIL) with subcutaneous injections of insulin (10 units/kg/day) and liraglutide (0.6 mg/kg/day). In previous studies, the dose of liraglutide ranged from 0.2 to 1 mg/kg, so we chose a relatively large dose of 0.6 mg/kg [16–18]. Although not expected, the rapid weight loss of >15-20% was defined as a potential humane endpoint for the study.

Body weight and blood glucose levels of pedal dorsal vein blood samples measured using Accu-Chek compact glucometer (Roche) were recorded once a week. At the end of the experiment, mice were killed with an overdose of isoflurane (5%) followed by decapitation. The trunk blood and liver tissues were collected from experimental animals, and serum was separated for analyzing C-peptide. All animal procedures were approved by the Institutional Animal Care and Use Committee at the Institute of Laboratory Animals Science, Chinese Academy of Medical Sciences, and Peking Union Medical College and conducted according to the Laboratory Animal Management Regulations in China and adhered to the Guide for the Care and Use of Laboratory Animals published by the National Institutes of Health [19]. All animal studies were performed in accordance with the Animal Research: Reporting of In Vivo Experiments guidelines [20].

### 2.2. Hematoxylin and Eosin Staining

Liver specimens were fixed in 4% paraformaldehyde overnight, embedded in paraffin, and cut into 5 *μ*m thick sections. Then, the slides were then stained with hematoxylin (cat. no. G1004-100ML; Wuhan Servicebio Technology Co., Ltd.) for 5 min at room temperature, followed by immersion in 1% ethanol hydrochloride for 5 min. After washing with water, the slides were stained with eosin (cat. no. G1001-100ML; Wuhan Servicebio Technology Co., Ltd.) for 5 min and dehydrated with an alcohol gradient. Five fields were randomly selected from each section separately and used to gauge hepatic steatosis (steatotic cell number/total cell number) on a scale of 0 to 4: 0: no obvious degeneration; 1: ≤25%; 2: 25~50%; 3: 50~75%; and 4: >75%.

### 2.3. TUNEL Staining

Liver sections were dewaxed with anhydrous ethanol and xylene, washed with distilled water, and then incubated in proteinase K working solution (cat. no. G1205; Wuhan Servicebio Technology Co., Ltd.) for 30 min at 37°C, followed by washing with phosphate-buffered saline (PBS) solution. The TUNEL reaction mixture (cat. no. G1501; Wuhan Servicebio Technology Co., Ltd.) was subsequently added and incubated at 37°C for 60 min. The sections were washed with PBS and then incubated with DAPI solution (cat. no. G1012; Wuhan Servicebio Technology Co., Ltd.) for 10 min at room temperature. After that, the specimens were washed with PBS and observed under a fluorescence microscope. Five fields of view were randomly selected on each slide, and the percentage of apoptosis-positive cells/total cells was calculated.

### 2.4. Periodic Acid-Schiff (PAS) Staining

Liver sections were stained with PAS reagent (cat. no. G1008-20ML; Wuhan Servicebio Technology Co., Ltd.) for 40 min at room temperature and then washed with 1% ethanol hydrochloric. After rinsing with distilled water, the sections were dehydrated with an alcohol gradient. Images were observed using a light microscope.

### 2.5. Liver Lipid Measurements

Hepatic triglycerides were extracted from frozen liver tissue and measured using a Triglyceride Content Assay Kit (cat. no. E1013; Applygen Technologies Inc.) according to the manufacturer's instructions. Values of TG were standardized to protein concentration.

### 2.6. Assessment of Lipid Peroxidation and Oxidative Stress Markers

Liver tissue was homogenized and then centrifuged at 12,000 × g for 15 min at 4°C. After extraction of the supernatant, the concentration of total protein was determined using a BCA Protein Assay Kit (cat. no. P0012S; Beyotime Institute of Biotechnology). Subsequently, malondialdehyde (MDA) levels were determined using an MDA kit (cat. no. S0131S; Beyotime Institute of Biotechnology), and the levels of antioxidant enzymes were determined using a superoxide dismutase (SOD) kit (cat. no. S0101S; Beyotime Institute of Biotechnology) and a glutathione peroxidase (GPX) kit (cat. no. S0056; Beyotime Institute of Biotechnology) according to the manufacturer's instructions.

### 2.7. Western Blot Analysis

Liver tissues were homogenized in RIPA lysis buffer (cat. no. P0013C; Beyotime Institute of Biotechnology) containing protease and phosphatase inhibitors (cat. no. P1045; Beyotime Institute of Biotechnology), and the supernatant was then centrifuged at 12,000 x g for 15 min at 4°C. A BCA Protein Assay Kit (cat. no. P0012S; Beyotime Institute of Biotechnology) was used to determine the protein concentration. Equal amounts (20 *μ*g each) of proteins were separated using 12% SDS-PAGE, transferred to polyvinylidene difluoride membranes, and then blocked with 5% nonfat milk for 1 h. The membranes were incubated with the following primary antibodies at 4°C overnight: rabbit anti-S33-phosphorylated (p)*β*-catenin (1 : 5,000; cat. no. 80067-1-RR; Proteintech Group, Inc.), *β*-catenin (1 : 5,000; cat. no. 51067-2-AP; Proteintech Group, Inc.), GSK-3*β* (1 : 1,000; cat. no. 22104-1-AP; Proteintech Group, Inc.), caspase-3 (1 : 1,000; cat. no. 9662S; Cell Signaling Technology, Inc.), Bcl-2 (1 : 1,000; cat. no. 12789-1-AP; Proteintech Group, Inc.), Bax (1 : 5,000; cat. no. 50599-2-Ig; Proteintech Group, Inc.), mouse anti-S9-pGSK-3*β* (1 : 1,000; cat. no. 67558-1-Ig; Proteintech Group, Inc.), and rabbit anti-*β*-actin (1 : 1,000; cat. no. 4970S; Cell Signaling Technology, Inc.) primary antibodies. After washing with TBST, the membranes were incubated with HRP-conjugated secondary antibodies against rabbit IgG (1 : 1,000; cat. no. 7074S; Cell Signaling Technology, Inc.) or HRP-conjugated secondary antibodies against mouse IgG (1 : 1,000; cat. no. 7076S; Cell Signaling Technology, Inc.) for 1 h at room temperature. *β*-Actin was used as the internal control. The resulting protein bands were visualized using an enhanced chemiluminescence (ECL) reagent (cat. no. P06M31M; Gene-Protein Link) according to the manufacturer's instructions, and densitometric analysis of protein bands was performed using ImageJ software (version 1.46a; National Institutes of Health).

### 2.8. Reverse Transcription-Quantitative Polymerase Chain Reaction (RT-qPCR)

Total RNA was extracted from liver tissue using Tissue Total RNA Isolation Kit V2 (cat. no. RC112; Vazyme Biotech Co., Ltd.) and was reverse-transcribed using PrimeScript™ RT Reagent Kit with gDNA Eraser (cat. no. RR047A; Takara Biotechnology Co., Ltd.). qPCR was performed using an ABI 7500 RT-PCR System (Applied Biosystems, Thermo, USA) and a TB Green® Premix Ex Taq™ II (cat. no. RR82LR; Takara Biotechnology Co., Ltd.). The sequence information of all primers used is listed in Table 1. The following thermocycling conditions were used for the qPCR: initial denaturation for 30 s at 95°C followed by 40 PCR cycles (95°C for 5 sec, 60°C for 30 sec, and 72°C for 30 sec). The relative gene expression was calculated using the 2^-*ΔΔ*Cq^ method [21] and normalized to the internal reference gene *β*-actin.

### 2.9. Statistical Analysis

All data analysis was carried out with SPSS 25.0 software (IBM Corp.). Data from at least three independent experiments are expressed as the mean ± standard error. Results were analyzed by one-way ANOVA with Bonferroni's post hoc test for statistical comparisons among more than two groups. Differences were considered statistically significant at *p* < 0.05.

## 3. Results

### 3.1. Effects of Liraglutide on Metabolic Parameters in DM Mice

In our previous study, we reported that STZ-induced diabetic mice exhibited hyperglycemia and a severe loss of body weight, whereas liraglutide treatment significantly decreased blood glucose levels in DM mice but did not restore weight loss. However, insulin monotherapy and the combination of the two drugs failed to control blood glucose and body weight well. There were no significant differences in serum C-peptide among the groups [22].

### 3.2. Effects of Liraglutide on Hepatic Lipid and Glycogen Accumulation in DM Mice

**Figure 1:**
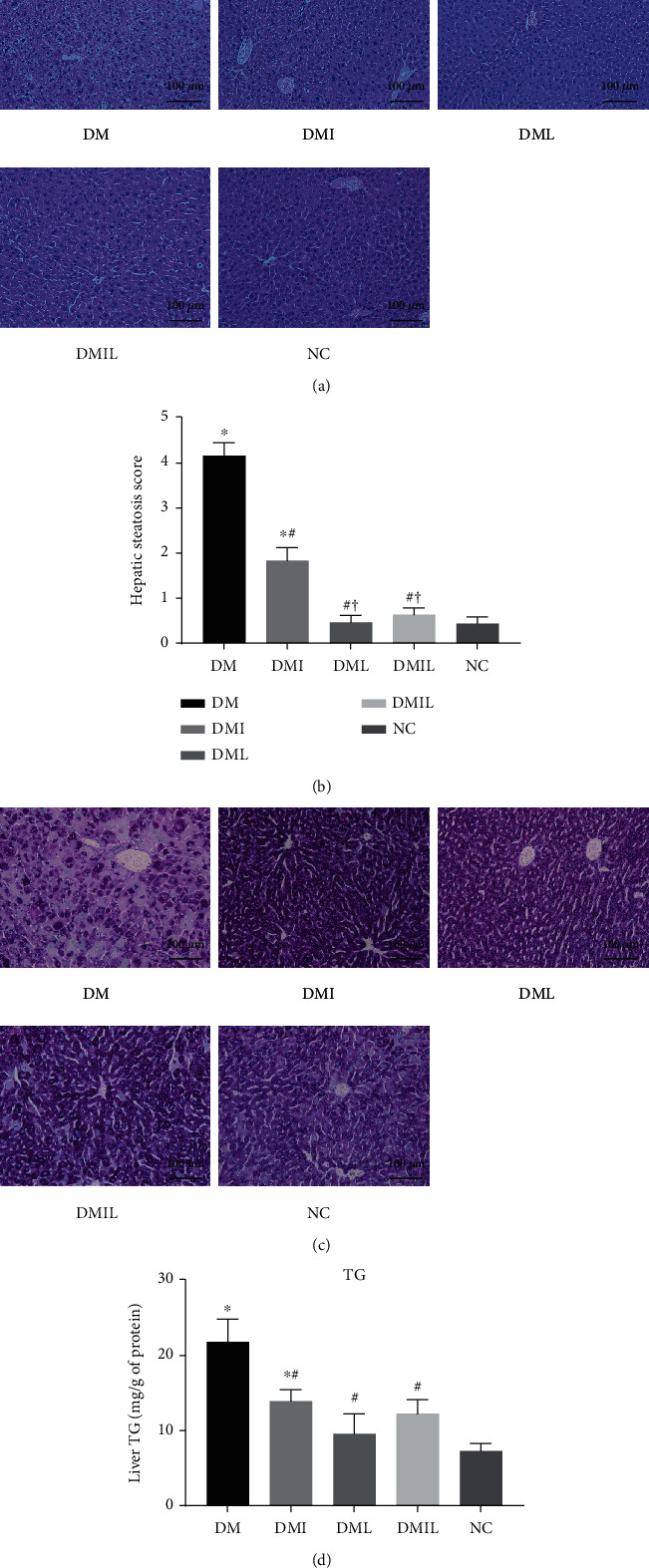
Effects of liraglutide on histological alterations in DM mice. (a) HE staining of liver tissue. Scale bar is 100 *μ*m in the images. (b) Hepatic steatosis score. (c) PAS staining of liver tissue. (d) Triglyceride content in the liver. Data are expressed as mean ± SD (*n* = 4/group); *p* < 0.05 was defined as statistically significant. DM: saline-treated type 1 diabetes group. DMI: insulin treatment group; DML: liraglutide treatment group; DMIL: insulin+liraglutide treatment group; NC: normal glucose tolerance group; ^∗^: compared to NC; #: compared to DM; †: compared to DMI; ‡: compared to DML.

Figure 1(a) shows the photomicrographs of hematoxylin and eosin-stained liver tissue sections of each group of mice. Compared with the NC group, the liver of the DM group exhibited significant cellular vacuolization and increased steatosis score (Figure 1(b)), accompanied by disorganized hepatic cords, dilated sinusoids, and infiltration of inflammatory cells. However, vacuolar degeneration in the liver was significantly decreased after insulin or liraglutide treatment. The improvement was more pronounced in the DML and DMIL groups compared to the DMI group. The DML and DMIL groups had normal hepatocyte appearance with a steatosis score comparable to that of the control group. The extent of PAS staining indicated that the accumulation of glycogen in the liver of DM mice was less than that in the control group, whereas insulin or liraglutide treatment led to an increase in the liver glycogen content (Figure 1(c)). The hepatic lipid content was also significantly higher in DM mice than in the NC group and decreased after insulin or liraglutide treatment (Figure 1(d)).

### 3.3. Liraglutide Reduced Hepatocyte Apoptosis in DM Mice

**Figure 2:**
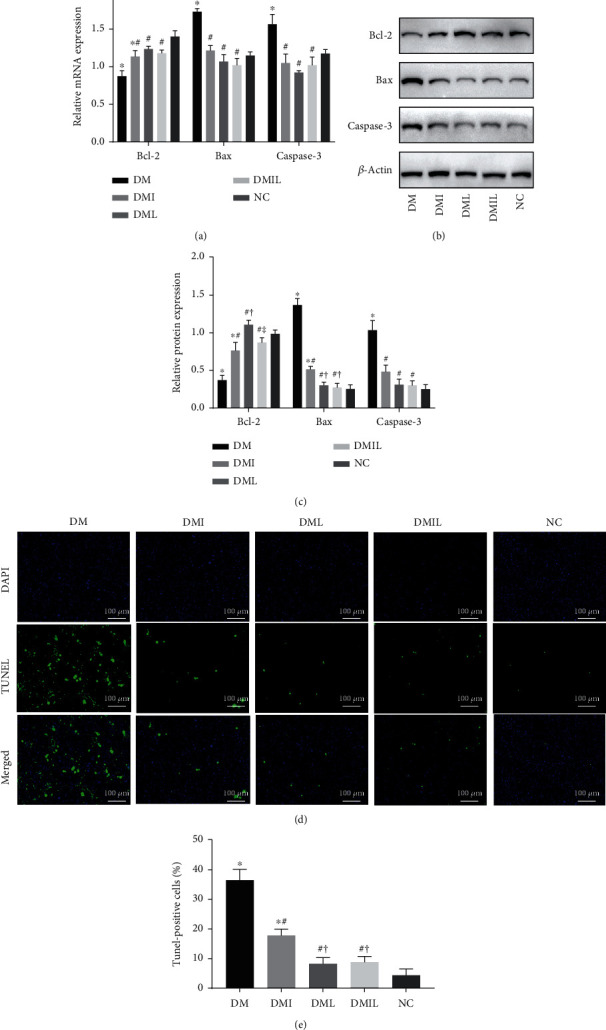
Effects of liraglutide on hepatocyte apoptosis in DM mice. (a) Relative mRNA expression of Bcl-2, Bax, and caspase-3 in liver tissues was assessed by quantitative RT-PCR. (b, c) Representative Western blot images and densitometry measurements of Bcl-2, Bax, and caspase-3 expressions in liver tissues. (d) Representative immunofluorescence images of TUNEL-stained liver tissue sections. (e) Percentage of TUNEL-positive cells. Data are expressed as mean ± SD (*n* = 4/group); *p* < 0.05 was defined as statistically significant. Scale bar is 200 *μ*m in the images. DM: saline-treated type 1 diabetes group; DMI: insulin treatment group; DML: liraglutide treatment group; DMIL: insulin+liraglutide treatment group; NC: normal glucose tolerance group; ^∗^: compared to NC; #: compared to DM; †: compared to DMI; ‡: compared to DML; Bax: Bcl-2-associated X protein; RT-qPCR: reverse transcription quantitative real-time polymerase chain reaction; TUNEL: terminal deoxynucleotidyl transferase- (TdT-) mediated dUTP nick-end labeling.

In order to assess hepatic apoptosis, the mRNA and protein expressions of Bax, Bcl-2, and caspase-3 were determined using RT-qPCR and Western blot analysis. Significantly increased expression levels of Bax and caspase-3 were observed in the liver of the DM group compared to the control group, with a decrease in the expression level of Bcl-2 (Figures 2(a)–2(c)). Interestingly, insulin or liraglutide administration significantly decreased the expression levels of Bax and caspase-3. TUNEL staining demonstrated a significant increase in the number of TUNEL-positive cells in liver tissue of DM mice compared with controls (Figures 2(d) and 2(e)). However, apoptotic cells were significantly reduced in the DMI, DML, and DMIL groups, and liraglutide exerted a more pronounced effect than insulin.

### 3.4. Effects of Liraglutide on Hepatic Oxidative Stress in DM Mice

**Figure 3:**
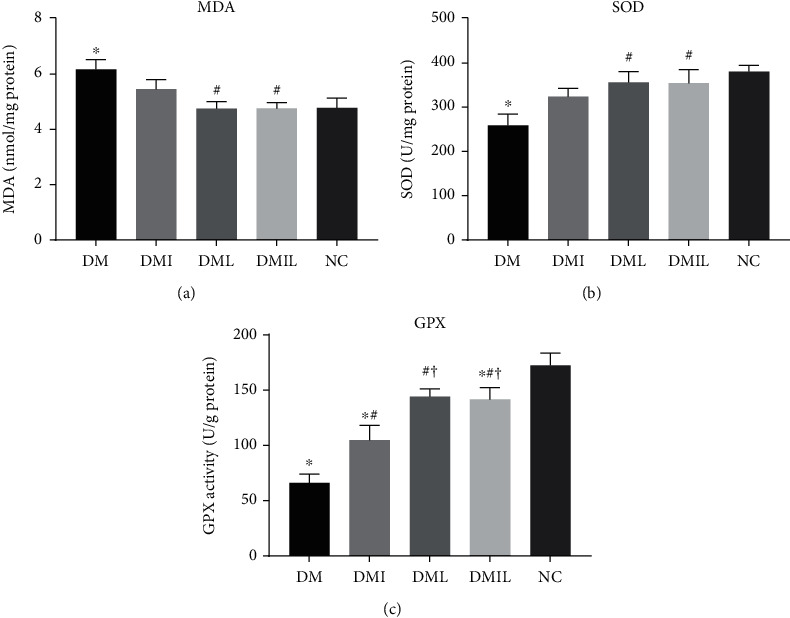
Effects of liraglutide on (a) MDA and hepatic oxidative stress markers (b) SOD and (c) GPX. Data are expressed as mean ± SD (*n* = 4/group); *p* < 0.05 was defined as statistically significant. DM: saline-treated type 1 diabetes group; DMI: insulin treatment group; DML: liraglutide treatment group; DMIL: insulin+liraglutide treatment group; NC: normal glucose tolerance group; ^∗^: compared to NC; #: compared to DM; †: compared to DMI; ‡: compared to DML.

Compared with the control group, DM mice had increased hepatic oxidative stress levels, as indicated by significantly increased MDA levels (Figure 3(a)). However, either insulin or liraglutide treatment reduced MDA levels, with liraglutide exerting a more prominent effect than insulin. Moreover, the activity of antioxidant enzymes such as SOD and GPX in the liver was decreased in the DM group compared to the control group (Figures 3(b) and 3(c)). However, either insulin or liraglutide treatment significantly increased the activity of SOD and GPX compared to the DM group, with liraglutide displaying a better effect than insulin.

### 3.5. Effects of Liraglutide on Hepatic Inflammation in DM Mice

**Figure 4:**
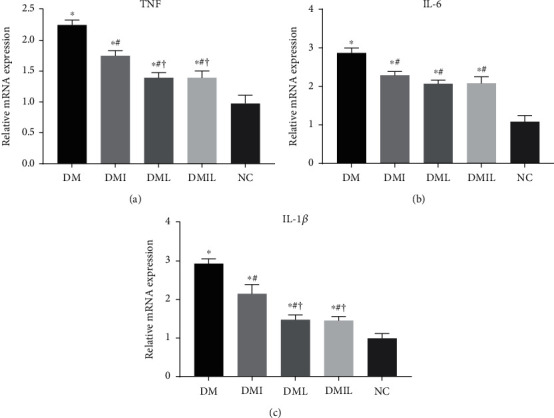
Effects of liraglutide on relative mRNA expression of (a) TNF, (b) IL-1*β*, and (c) IL-6. Data are expressed as mean ± SD (*n* = 4/group); *p* < 0.05 was defined as statistically significant. DM: saline-treated type 1 diabetes group; DMI: insulin treatment group; DML: liraglutide treatment group; DMIL: insulin+liraglutide treatment group; NC: normal glucose tolerance group; ^∗^: compared to NC; #: compared to DM; †: compared to DMI; ‡: compared to DML.

DM mice showed evidence of an obvious inflammatory state compared to controls, as indicated by significantly increased mRNA levels of TNF-*α*, IL-6, and IL-1*β* (Figures 4(a)–4(c)). However, all these changes were significantly reversed after insulin or liraglutide treatment, with significantly decreased expression levels of hepatic TNF-*α*, IL-6, and IL-1*β* in the DMI, DML, and DMIL groups compared with the DM group. In addition, liraglutide showed a more pronounced effect of decreasing IL-1*β* and TNF-*α* expressions than insulin.

### 3.6. Liraglutide Activates the Wnt/*β*-Catenin Signaling Pathway in the Liver of DM Mice

**Figure 5:**
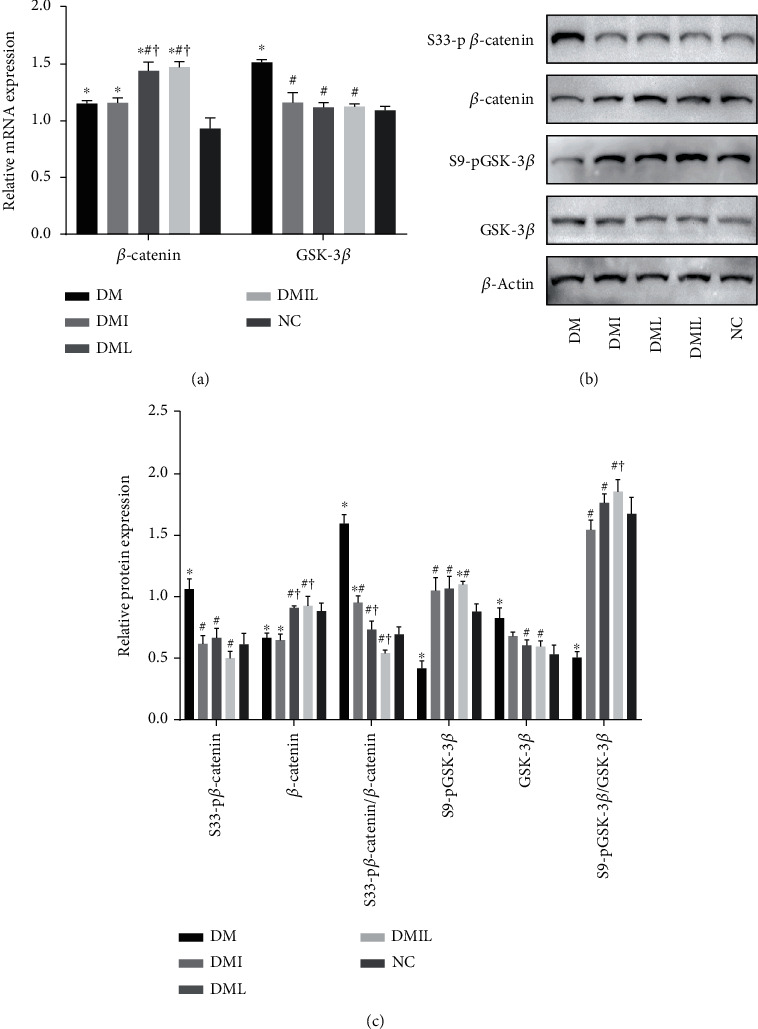
Effects of liraglutide on Wnt/*β*-catenin pathway activity in DM mice. (a) Relative mRNA expression and quantification of Wnt/*β*-catenin signaling pathway-related molecules. (b, c) Protein bands and relative protein expression of Wnt/*β*-catenin signaling pathway-related molecules were detected by Western blot analysis; *β*-actin was included as a reference for normalization. Data are expressed as mean ± SD (*n* = 4/group); *p* < 0.05 was defined as statistically significant. DM: saline-treated type 1 diabetes group; DMI: insulin treatment group; DML: liraglutide treatment group; DMIL: insulin+liraglutide treatment group; NC: normal glucose tolerance group; ^∗^: compared to NC; #: compared to DM; †: compared to DMI; ‡: compared to DML.

Gene and protein expressions of the Wnt/*β*-catenin signaling pathway in the liver of mice are presented in (Figure 5). The DM group showed a significant reduction in phosphorylation of GSK-3*β* along with a pronounced increase in phosphorylation of *β*-catenin in the liver compared to the control group. Treatment with liraglutide or insulin effectively modulated this signaling pathway as evidenced by increased protein expression levels of pGSK-3*β* and decreased expression of p*β*-catenin and GSK-3*β* compared to the DM group. Furthermore, liraglutide treatment enhanced the mRNA and protein expression levels of *β*-catenin compared with the DM and DMI groups. These results suggested that both liraglutide and insulin upregulated the Wnt/*β*-catenin signaling pathway in the liver of T1DM mice, with liraglutide having a more prominent effect than insulin.

## 4. Discussion

T1DM is caused by autoimmune destruction of *β*-cells in the pancreas, resulting in hyperglycemia and a progressive decline in insulin secretion. Hepatic injury and dysfunction have been reported in patients with T1DM during the disease course [23, 24], and an increasing number of studies have shown that liraglutide not only lowers blood glucose levels but also has hepatoprotective effects in patients with NAFLD [25, 26]. To the best of our knowledge, this is the first study to compare the effects of peripherally administered liraglutide, insulin, and the combination on liver injury in a mouse model of STZ-induced T1DM and explore the underlying mechanisms. The results of the present study showed that liraglutide might ameliorate T1DM-related liver injury by improving inflammation and oxidative stress and reducing apoptosis.

In this study, T1DM impaired liver function, as evidenced by increased hepatic steatosis, apoptosis, and lipid accumulation as well as decreased glycogen content in the DM group compared to that in the control group. As biomarkers of inflammatory response, significantly increased levels of TNF-*α*, IL-6, and IL-1*β* were found in liver tissues of T1DM mice. MDA, a marker of lipid peroxidation, was increased in the liver of T1DM mice, whereas the activity of antioxidant enzymes such as SOD and GPX was decreased. All these liver abnormalities are demonstrated by previous studies related to animal models of T1DM [27–31]. Furthermore, in previous clinical studies, T1DM patients showed decreased blood glutathione and cysteine, along with increased oxidative stress and lipid peroxidation [2], suggesting that oxidative stress or shortage of antioxidant defense systems and further activation of the inflammatory cascade might be the underlying mechanism of liver injury and dysfunction.

Interestingly, insulin treatment showed a significant improvement in liver damage in STZ-induced T1DM mice in the present study. Insulin is the primary treatment for T1DM, and previous studies have shown that insulin may prevent liver cell apoptosis by reducing the effects of hydroxyl radicals [32] or by inhibiting the activity of c-Jun NH2 terminal kinase [33]. We demonstrated that insulin significantly reduced caspase-dependent hepatocyte apoptosis and decreased oxidative stress and inflammatory factor expression in hepatocytes. In addition, insulin significantly ameliorated hepatic steatosis and increased the glycogen content in the liver of T1DM mice.

Notably, GLP-1 was demonstrated to be beneficial for hepatocyte apoptosis in a model of NAFLD [7]. It has been reported that liraglutide reduces hepatocyte apoptosis by activating the NRF2 signaling pathway to enhance the antioxidant activity of hepatocytes [8]. Moreover, liraglutide reduced levels of reactive oxygen species and inflammatory factors such as IL-1*β*, IL-6, and TNF-*α* in a high-fat diet-induced liver injury model [34], whereby IL-1*β* and TNF-*α* have been demonstrated to reduce insulin biosynthesis and induce apoptosis [35] and are also important factors in insulin resistance in T2DM and hepatogenic diabetes [36]. In our study, we found that liraglutide treatment alone or in combination with insulin not only reduced hepatocyte apoptosis, inflammation, and oxidative stress to improve T1DM-related liver injury but also showed better efficacy than insulin. However, the hepatic metabolism effect of GLP-1 may involve other pathways [37], as the GLP-1 receptor was not detected in the liver [38].

To further explore the mechanistic pathways underlying the promising therapeutic benefits of liraglutide and insulin, we investigated their effects on the Wnt/*β*-catenin signaling pathway. The Wnt/*β*-catenin signaling pathway in the liver has been reported to be involved in regulating cell apoptosis, proliferation, and glycolipid metabolism [39, 40]. For example, activation of the Wnt/*β*-catenin pathway promoted hepatocyte proliferation and attenuated inflammation and apoptosis in mice with liver ischemia-reperfusion injury [13], whereas inhibition of this pathway may aggravate carbamazepine- or lipopolysaccharide-induced liver damage [41, 42]. Furthermore, the expression level of *β*-catenin, the most critical cofactor in the typical Wnt/*β*-catenin pathway, has been shown to be significantly decreased in animal models of liver injury, and its activation may attenuate oxidative stress and apoptosis in the liver by regulating cellular redox homeostasis [12, 13]. However, the role played by the Wnt/*β*-catenin pathway in liver damage of T1DM animal models has not been reported. Previous studies have demonstrated that insulin inhibits liver gluconeogenesis by inhibiting FoxO1 which allows *β*-catenin to bind TCF7L2 [43] and also activates hepatic Wnt/*β*-catenin signaling through stearoyl-CoA desaturase 1 and porcupine [44]. Qin et al. demonstrated that the ameliorative effect of liraglutide on hepatic insulin resistance in db/db mice was mediated by the Wnt/*β*-catenin signaling pathway [14], whilst exendin-4 reduced oleic acid-induced steatosis in HepG2 cells via the Wnt/*β*-catenin signaling pathway [45]. In this study, we investigated for the first time whether the application of liraglutide and insulin could attenuate liver injury in diabetic mice by activating the Wnt/*β*-catenin signaling pathway, and the results suggested that either liraglutide, insulin, or the combined drugs led to a significant increase in expression ratios of pGSK-3*β*/GSK-3*β* and a decrease in S33-p*β*-catenin/*β*-catenin. Therefore, liraglutide and insulin may regulate glucolipid metabolism and attenuate oxidative stress and inflammatory state in the liver of T1DM mice by activating the Wnt/*β*-catenin signaling pathway, thereby ameliorating hepatocyte degeneration and apoptosis.

However, the present study has a few limitations. First, biomarkers of liver function were not measured due to limited serum specimens, but detailed pathological data also provide an adequate description of the liver damage. Second, we measured blood glucose rather than glycosylated hemoglobin, which may not completely reflect changes in blood glucose. We hypothesize that insulin monotherapy and combination therapy may have glycemic control effects, as demonstrated by the reduced deaths of mice due to hyperglycemia, although there was no significant improvement in blood glucose values in these two groups. Finally, this study lacked a comparison of groups treated with activators or antagonists of the Wnt signaling pathway, and more experimental data are needed to further elucidate the specific mechanisms by which liraglutide exerts its hepatoprotective effects.

## 5. Conclusion

In conclusion, the results of the present study suggested that liraglutide, insulin, and their combination improved hepatic inflammation, oxidative stress status, and apoptosis in an STZ-induced T1DM mouse model, which appears to be associated with activation of the Wnt/*β*-catenin signaling pathway. In addition, liraglutide showed better capacity in alleviating liver injury than insulin. The present study provides new evidence for the protective effect of liraglutide on T1DM-related liver injury, which has important implications for the treatment of NAFLD in patients with T1DM.

## Figures and Tables

**Table 1:** Primer sequence for RT-qPCR.

Gene	Forward primers (5′-3′)	Reverse primers (5′-3′)
GSK-3*β*	GTAGCCCAGGGAGGTCACTA	CAGCCTTCCTAAGCTGGCAT
*β*-Catenin	CTGGGACTCTGCACAACCTT	CAGTGTCGTGATGGCGTAGA
Bax	TCTCCGGCGAATTGGAGATG	ACCCGGAAGAAGACCTCTCG
Bcl-2	GCAGCTTCTTTTCGGGGAAG	CTCCAGCATCCCACTCGTAG
Caspase-3	TGGCTTGCCAGAAGATACCG	ATGCTGCAAAGGGACTGGAT
TNF	AACTTCGGGGTGATCGGTCC	GTGTGGGTGAGGAGCACGTA
IL-6	ACTTCACAAGTCGGAGGCTT	TGCAAGTGCATCATCGTTGT
IL-1*β*	AAGGAGCTATCACTTGACCAC	AAGCTGGATGCTCTCATCAGG

## Data Availability

The datasets used and/or analyzed during the current study are available from the corresponding authors on reasonable request.
